# Country-Specific Roadmaps for Scaling Up Integrated Care in Belgium, Slovenia, and Cambodia – Lessons Learned from the SCUBY Project

**DOI:** 10.5334/ijic.8618

**Published:** 2024-12-20

**Authors:** Martin Heine, Monika Martens, Daniel Boateng, Grace Marie Ku, Roy Remmen, Edwin Wouters, Srean Chhim, Por Ir, Antonjia Poplas Susič, Wim van Damme, Josefien van Olmen, Kerstin Klipstein-Grobusch, on behalf of the SCUBY consortium

**Affiliations:** 1 Julius Global Health, Julius Center for Health Sciences and Primary Care, University Medical Center Utrecht, Utrecht University, Utrecht, Netherlands; 2 Institute of Sport and Exercise Medicine, Faculty of Medicine and Health Sciences, Stellenbosch University, Cape Town, South Africa; 3 Department of Public Health, Institute of Tropical Medicine, Antwerp, Belgium; 4 Department of Family Medicine and Population Health (FAMPOP), Faculty of Medicine and Health Sciences, University of Antwerp, Belgium; 5 Department of Epidemiology and Biostatistics, Kwame Nkrumah University of Science and Technology, Kumasi, Ghana; 6 Department of Family Medicine and Population Health (FAMPOP), Faculty of Medicine and Health Sciences, University of Antwerp, Belgium; 7 Centre for Population, Family & Health, Department of Social Sciences, University of Antwerp, Antwerp, Belgium; 8 Centre for Health Systems Research & Development, University of the Free State, Bloemfontein, South Africa; 9 National Institute of Public Health, Phnom Penh, Cambodia; 10 Ljubljana Community Health Centre, Ljubljana, Slovenia; 11 Department of Family Medicine, Faculty of Medicine, University of Ljubljana, Ljubljana, Slovenia; 12 Division of Epidemiology and Biostatistics, School of Public Health, Faculty of Health Sciences, University of the Witwatersrand, Johannesburg, South Africa

**Keywords:** integrated health care systems, diabetes, hypertension, scale-up, reciprocal learning

## Abstract

**Introduction:**

The SCUBY project aimed to provide knowledge on the scaling-up of an Integrated Care Package (ICP) for type 2 diabetes and hypertension across three distinct health systems (Cambodia, Slovenia, and Belgium). Here, we analyse the different elements of the country-specific scale-up roadmaps to identify similarities and differences, and share lessons learned.

**Methods:**

Thematic analysis was used to derive crucial roadmap elements from key SCUBY documents (n = 20), including policy briefs, interim reports, research outputs, and consortium meeting notes.

**Results:**

Roadmap elements differed according to priority needs, features of the (health) systems, and partly reflected the position of the SCUBY research team within each country. Common cross-country elements were: task-shifting to patients themselves, nurses and community health workers; strengthening monitoring and evaluation; and creating an enabling environment for ICP implementation.

**Discussion:**

Scale-up of complex interventions requires continuous engagement of multiple stakeholders and contextualization of action plans. The linkage of research teams with key implementation stakeholders and policy makers creates change-teams, allowing advancement from formative research to implementation of roadmap strategies and full scale-up in due time.

**Conclusion:**

The development processes and contents of the roadmaps provided essential and reciprocal learnings. These learnings help shape future policy dialogues and best practices to tackle chronic disease in each participating country.

## Introduction

Despite a rapidly increasing burden of non-communicable diseases (NCDs) globally, particularly in low-income populations [1], a large part of the world’s population lacks access to adequately integrated and comprehensive health care services and strategies that are inclusive across the patient demographic [2]. Type 2 diabetes (T2D) and hypertension (HT) are of particular relevance due to their shared risk factors, relationship to social determinants of health, and their co-occurrence [3]. According to 2019 global estimates, 463 million adults live with T2D, and 1.13 billion with HT [4].

Effective interventions for integrated treatment and control of both T2D and HT are available and cost-effective and include the following overall elements: (a) early detection and diagnosis, (b) treatment in primary care services, (c) health education, (d) self-management support to patients and caregivers, and (e) collaboration between caregivers [5,6]. These bundled interventions can be identified as an ‘integrated care package’ (ICP), and have been shown to be feasible and effective in mitigating cardiovascular disease and broader health risks [7,8]. The ICP is in line with various chronic care models and WHO guidelines on integrated care and essential interventions for diabetes and hypertension [9]. However, successfully scaling up an ICP package for T2D and HT can be challenging [5].

The aim of the “SCale-Up of integrated care for diaBetes and hYpertension in Cambodia, Slovenia and Belgium” (SCUBY) project was to develop, implement, and evaluate country-specific, evidence-based roadmap strategies to support the scaling up of diabetes and hypertension care in each country [5,10]. Within the SCUBY project, a three-dimensional framework for scale-up has been described. Action along one or more of these dimensions can be considered as “scale-up”:

- increasing population coverage
- expanding the intervention package; and
- integration of the ICP into the health system.

One of the key objectives for the SCUBY project was to generate lessons across contexts on the scale-up strategies for integrated care for T2D and HT such to better understand pathways to scale-up in each country.

In the present paper, we (i) describe the contents and distinct elements of the scale-up roadmaps in our three case study countries and (ii) reflect on cross-contextual learnings about roadmaps from these three cases. Aligned with these objectives, our research questions are: (1) What do the country-specific scale-up roadmaps look like (at the end of the SCUBY project), what actions and strategies do they entail?; and (2) What are reciprocal, cross-country lessons we can draw from them, in particular about their similarities and differences?

## Research methods

### Study design

The SCUBY project employed a multiple case study quasi-experimental design to develop, implement, and evaluate strategies for the scale-up of integrated care for T2D and HT. A comprehensive protocol for the SCUBY project has been reported elsewhere [5]. The SCUBY project entailed three phases: formative, intervention, and evaluation.

– A situational analysis was conducted during the formative phase to assess ICP implementation (baseline) and identify multi-level barriers and facilitators across the three countries.
– Subsequently, during the intervention phase, the learnings from the formative phase were amalgamated into country-specific “living” (i.e., evolving) roadmaps; defined as “an action plan delineating the targets, planning, and progression of scale-up strategies, identifying actors, actions, and timelines based upon priorities in place and time” [5]. In all three countries, the scale-up activities aim to improve integrated primary care [11].
– The evaluation phase in SCUBY included four types: process, scale-up, cost, and impact evaluations [11]. This article is part of the process evaluation, focusing on the implementation of roadmaps for scaling up integrated care.

### Study settings

**Figure 1:**
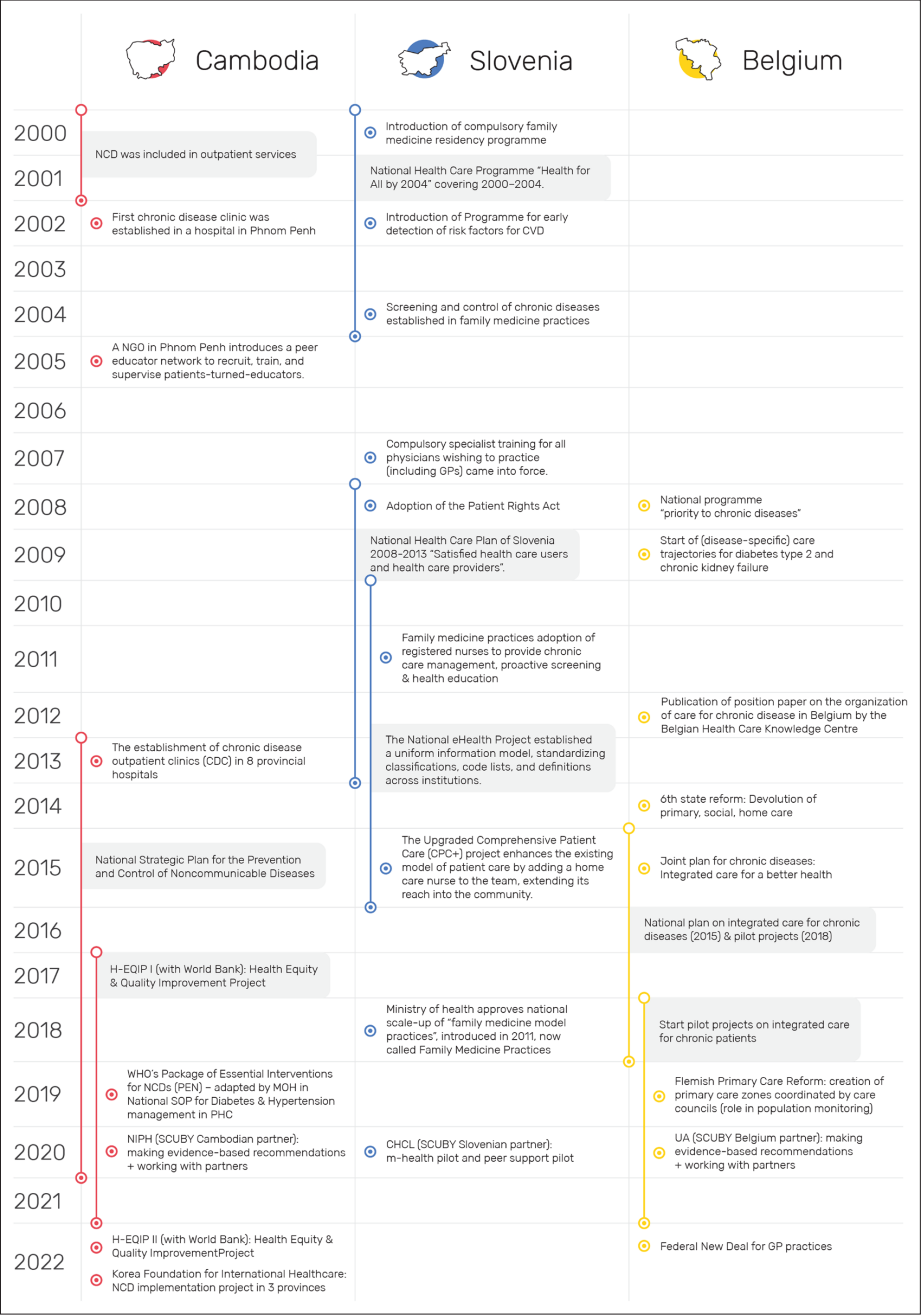
Key health system developments and policy initiatives relevant to the scale-up of integrated care prior to and during the SCUBY project. NCD, Non-Communicable Disease; CVD, Cardiovascular Disease; MHO, Ministry of Health; NGO, Non-governmental Organisation; GP, General Practitioner; H-EQIP, Health Equity and Quality Improvement Project; NIPH, National Institute of Public Health; UA, University of Antwerp; CHCL, Community Health Center Ljubljana. Reference to position paper (Belgium) [21].

While each country provided unique insights, this cross-country project also highlights the opportunity to learn from similarities and differences across diverse contexts. Health system characteristics and existing scale-up strategies (see online supplement and Figure 1) influence roadmap development and implementation.

**Cambodia** is a lower-middle income country of about 15.3 million inhabitants (2019) with an annual growth rate of 1.2% [12]. Importantly, Cambodia is undergoing an epidemiological transition with the emerging prominence of NCDs. In 2016, 24% of total deaths in Cambodia were attributed to cardiovascular disease [13]. Furthermore, the prevalence of T2D in 2016 was reported at 9.6% among adults aged 18–69 years; an increase from 2.9% among adults aged 25–64 years in 2010 [4,14]. Conversely, its annual health spending on NCDs in 2019 was about 113 USD per capita relative to an overall health expenditure of 321 (USD/capita) in 2019; an increase of ~30% in five years [15]. A population-based survey conducted in Cambodia as part of the SCUBY project’s formative phase suggested a prevalence of 35.2% for hypertension and 11.0% for T2D among those aged 40 years or over [14,16]. Notably, of the 35.2% of people with hypertension identified, only 35.8% and 10.7% had good control of their blood pressure and glucose level, respectively [14,16]. This highlights that people with HT and T2D are often lost in the care process. It underscores the need for an ICP to enhance the health system’s response to HT, T2D, and other NCDs. The Ministry of Health has prioritized the WHO PEN interventions across 103 health districts. However, challenges remain in delivering quality care and regulating the expanding private sector [5]. Simultaneously, efforts to strengthen the rather marginalised public health system are being undertaken by the government with donor support [5].

**Slovenia** is a Central European, high-income country of about two million inhabitants. Its centralised national health system can be described as a combination of the Beveridge and Bismarck models with the main principles of universal coverage, solidarity, fairness in financing, non-profitability and equity in access for all groups of population [17]. In 2016, the mean annual spending on T2D was 2608 USD per person per year. All permanent residents of Slovenia are included in compulsory health insurance at the National Insurance Institute; almost 95% of population has, in addition, a voluntary complementary insurance. Since 2011, the government has invested in the scale-up and upgrading of primary care (family medicine) practices for chronic disease management [17,18]. This, amongst others, has standardised the diagnosis and management of patients with chronic disease. However, decreasing state expenditure on social services such as pensions has led to concerns for vulnerable groups, such as elderly people and minority groups. Community nurses have been used as a viable strategy to reach vulnerable populations.

**Belgium** is a high-income country of about 12 million inhabitants with a partially decentralised health care system. Furthermore, the organisation of Belgian healthcare and policy is influenced by non-governmental stakeholders including health professional organisations and private, not-for-profit associations of sickness funds. People have a free choice of health insurance and healthcare providers. Subsequently, both patients and healthcare providers have a relatively large decision space related to taking up the ICP. In Belgium, chronic diseases account for at least 90% of the societal burden of disease including disability, and substantial mortality; evidence from 27 European countries suggests that nearly one third of those over 15 years lives with multiple long-term conditions [19]. The high prevalence of multiple chronic conditions has sparked the need for and political commitments to integrated care, which have led to the development of various federal and federated policies since 2008 [20]. Yet the partial decentralisation of health care within the Belgium context has led to fragmentation of decision power which undermines these efforts towards integrated care [20].

Figure 1 provides a historical overview of health system developments and NCD policies, outlining the context for the SCUBY project and its roadmap development and implementation. A policy mapping is an important process evaluation tool [11] for scale-up to consider the contributions of previous and ongoing scale-up efforts, potential synergies and partners. Figure 1 events were initially identified during the situational analysis and later expanded through ongoing process evaluation, including document reviews and key informant interviews. The final mapping was verified with the research team near project completion, highlighting the dynamic policy context shaping the roadmaps.

### Data sources and analysis

To distil the components of the three roadmaps, we used various analytical approaches. First, we outlined key thematic elements based on discussions within the country teams and SCUBY consortium. Second, relevant documents from each country were identified and thematically analysed by an independent member (MH). Ten core roadmaps components as described by Weber et al. were identified during this qualitative analysis to organise the different actions and strategies [22]. A total of 20 key documents, in itself reflecting primary SCUBY data, were purposefully selected by the research team for document review and analysis, including technical reports to the European Union (n = 5), consortium meeting reports and minutes (n = 6), policy briefs (n = 3), pilot study protocols and reports (considered as “roadmaps” for Slovenia; n = 2), and “living” actual roadmap documents for Belgium (n = 2) and Cambodia (n = 2). Third, we subsequently simplified the complex outcome of the qualitative analysis using the World Health Organisation ExpandNET framework [23,24]. Finally, the analysis was presented to country leads and the SCUBY steering committee at the SCUBY colloquium and closing meeting (May 2023) for reflection on findings [23,24]. A reciprocal learning approach was used, fostering a collaborative exchange where each partner acted as both learner and coach [25].

## Results

The thematic analysis rendered a total of 343 codes pertaining to actions and strategies identified as part of the roadmap for integrated care across all three countries. Unique strategies and actions are in Online Supplement 2. Table 1 provides an overview of key roadmap components, with further details in Online Supplement 1 (ExpandNET framework). A narrative summary of activities, strategies, and actions for each country is presented here.

**Table 1:** Key roadmap actions and strategies to scale-up integrated care across the three participating countries.


CAMBODIA	SLOVENIA	BELGIUM

**Component 1: Health Service Delivery and Governance** Strategy 1.1: Increasing coverage of second-version PEN in primary healthcare. Strategy 1.2: Strengthening the workflow of Second-version PEN at the operational district level. Strategy 1.3: Revising/updating the components of ICP. Strategy 1.4: Adding community-based intervention to ICP. **Component 2: Medicine Supply** Strategy 2.1: Strengthening and updating the essential medicine supply system. Strategy 2.2: Reinforcing the capacity of staff in managing medicine inventories. **Component 3: HR** Strategy 3.1: Strengthening leadership and management of human resources for health at the operational district and health centre levels. Strategy 3.2: Ensuring appropriate staff/staff capacity/skills-mix through practical training on T2D & HT care (on-site training), including nurses and midwives. **Component 4: Health financing** Strategy 4.1: Increasing investments in T2D and HT. Strategy 4.2: Increasing service accessibility at public healthcare facilities. Strategy 4.3: Reducing financial burden to T2D and HT patients. **Component 5: Health information system** Strategy 5.1: Monitoring and evaluation.	1. An **m-health** intervention to support and empower patients (telemedicine). 2. A **group education** programme **by patients** (patients as educators). 3. **Community-based education** programme (with healthy lifestyle intervention(s)). 4. An **intra-team collaboration** project: developing clinical pathways of patients for better team management (with a focus on the education of registered nurses).	**1. Change management at practice (micro) level:** 1a: Better care for chronic conditions by GPs through training. 1b: Human resource management: Budget for nurse in primary care team. **2. Data monitoring at organisational/population (meso) level:** 2a: Monitoring of chronic care indicators in Primary Care Zones. 2b: Monitoring care organisation at practice level **3. Health financing at political (macro) level:** 3a: Budget for chronic care that stimulates quality. 3b: Alternative financing models in primary care.


HRH, human resources for health; HT, Hypertension; ICP, Integrated Care Package; PEN, Package of Essential Interventions; T2D, Type 2 Diabetes.

### Roadmap to scale up ICP in Cambodia

The Cambodian roadmap addresses two main concerns: improving the low performance of T2D and HT interventions in primary healthcare, specifically the “Package of Essential Non-Communicable Disease Interventions” (PEN) and increasing the proportion of individuals aware of their T2D and/or HT status. Many patients seek care in the private sector, leading to poor health outcomes and high out-of-pocket costs.

To address these two main issues, the Cambodian roadmap emphasizes strengthening of and further adapting WHO PEN (2nd version) implementation for NCDs and the need for broader public sector health system strengthening. It aligns with the WHO health system building blocks, targeting improvements in 1) health service delivery & governance, 2) medicine supply, 3) human resources for health, 4) health financing, and 5) health information system. Contextual adaptation of PEN will enable broader service coverage and integration, crucial for scaling up. Initial PEN-related activities included updating standard operating procedures and training on essential medicines, but these require dedicated government funding for NCDs and social health protection. Therefore, increasing investment in health financing is a key focus of the roadmap.

In addition to enhancing PEN integration, the roadmap emphasizes optimizing referral pathways among hospitals, health centres, and community health workers. Operational districts are encouraged to lead improvements in the referral system, with designated focal persons facilitating collaboration and communication. The roadmap also highlights the vital role of community health workers and peer networks in early detection and continuity of care. To support integrated care in Cambodia, (re)training needs must be identified at all levels, from healthcare workers to management. Implementing top-down decisions can scale up integrated care through performance-based bonuses, reduced out-of-pocket costs, and formalized funding for outreach activities related to T2D and HT. Additional investment in a monitoring and evaluation database is also essential for tracking patient populations and addressing care issues.

### Roadmap to scale-up ICP in Slovenia

The Slovenian roadmap revolves around a series of (ongoing) pilot studies that explored the feasibility and effectiveness of various models of task-shifting to promote self-management in vulnerable T2D and HT populations (e.g., elderly, rural populations). The strategies identified to enhance integrated chronic disease care included an m-health pilot intervention for vulnerable T2D/HT patients, a peer support program with patients as educators, a community-based healthy lifestyle initiative, and an intra-team collaboration project for primary care providers. These strategies were developed through multi-level stakeholder engagements, literature reviews, assessments using the ICP grid, evaluations of facilitators and barriers from patients’ perspectives, and health-economic surveys. The findings highlighted the need to build skills and knowledge to promote self-management for prevention and health promotion. The identified needs were operationalised into four interventions, consisting of two longer-running (m-health and peer support) pilot studies to provide evidence for future scale-up [26,27]. Strengthening the training and role of peer supporters, along with implementing task-shifting aids like m-health and telemedicine, are key components of the Slovenian roadmap.

### Roadmap to scale-up ICP in Belgium

The Belgium roadmap for the scale-up of integrated care focussed on a networking approach to facilitate dialogue, synergies, and collaboration between stakeholders including those in health funding, healthcare provision, research, and education space. This networking approach fits the fragmented nature of the Belgium health care system, and the scope of other ongoing activities within the country. Three key topics at various levels of the health system were identified as key to progress scale-up of integrated care for chronic conditions in Belgium: 1) change management at health care practice (micro) level, 2) data monitoring at population (meso organisational) level, and 3) health financing at the policy and political (macro) level.

The research team and stakeholders determined that promoting change management toward chronic care organization is essential, emphasizing interdisciplinary collaboration. This entails developing mechanisms for integrated services within existing structures, such as supporting GPs in chronic care through training programs and advocating for an expanded role for primary care nurses (micro-level). Various activities (meso-level) were conducted to support stakeholders in effectively using aggregate population health data on integrated care for T2D/HT in Belgium. These included establishing a Flemish working group, connecting different data sources, developing a dashboard for monitoring key indicators, identifying patient perspectives on integrated care, and assessing the health-economic implications of integrated care at facilities. At macro-level, the Belgian SCUBY roadmap advocated – in line with stakeholders’ call – for a broader policy reform towards a mixed provider payment model within primary care that stimulates quality, i.e., pay-for-quality. This would be a model where the benefits and drawbacks of the predominant fee-for-service provider payment system in Belgium are balanced out with those of a capitation provider payment system. In 2022, one of the Belgian SCUBY team members became a part of the working group on the Federal New Deal for GP practices (see Figure 1), a policy with this provider payment reform as one of its core themes.

### Cross-country reciprocal learnings

In line with the secondary aim of this paper, we identified similarities and differences between the scale-up roadmaps of the three countries.

Similarities relate to: (a) the roadmap content, and specifically several overarching strategies; and (b) boundary spanning skills gained by the change team (i.e. researchers and stakeholders involved in roadmap implementation for scale-up of integrated chronic care). With regards to *roadmap content*, similar strategies in each country’s roadmap were:

- task-shifting to decentralise integrated care through the involvement of community health workers (in Cambodia), patients (as peer supporters in Slovenia), and primary care nurses (in Belgium),
- strengthening monitoring and evaluation (as broader roadmap themes in Belgium and Cambodia; via mHealth in Slovenia), and
- supporting an enabling environment for implementation of the ICP (through broad health system strengthening in Cambodia, developing peer support networks to strengthen self-management in Slovenia; and via health financing mechanisms in Belgium).

As indicated between parentheses, the actions required to contribute to these strategies varied significantly. Nevertheless, these commonalities highlight the required ongoing work to address structural gaps as well as the relevance of human resources for health and the goal (and trend) of bringing integrated care closer to patient.

With regards to the *change team*, capacity building processes have been important as they have important effects. Organising and attending various policy dialogues on scaling up integrated care for T2D and HT presented opportunities to the research teams to build boundary spanning skills. Boundary spanning refers to practices of “reaching across borders, margins, or sections to build relationships, interconnections and inter- dependencies in order to manage complex problems” [28], and is key to involve multi-level and multi-sectoral stakeholders in scale-up efforts to drive collective action [29]. People with such boundary spanning skills are deemed important knowledge brokers and can act as moderators when looking for consensus across various interest groups. The time investment by the research team in strengthening relationships with and across stakeholders resulted in large resource teams or change teams for different strategies in the roadmap. In other words, the linkage of research teams with key implementation stakeholders and policy makers not only creates familiarity and trust, but can also create change teams, allowing advancement from formative research to implementation of roadmap strategies and full scale-up in due time. Each SCUBY country team had at least one member – often a professor or senior researcher – with a large network across health actors. As the project progressed and more stakeholder interviews as well as policy dialogues were organised, also the younger team members gained boundary spanning skills and were able to tap into that wider network. This increased the recognition of the research project and the SCUBY team in the various settings. This reciprocal learning highlights the importance of normative integration aspects [30], including having informal contacts and reputation building for the purpose of building alliances for more sustainable policy change.

Differences between the three scale-up roadmaps relate to: (a) the scope and format of the roadmap; (b) the scale-up dimension given priority to; and (c) the mandate of the change team. In addition to the diversity in actions and strategies, also the *scope and format* of each roadmap varied. In other words, the type of ‘document’ can come in different shapes and sizes. For example, while the *scope* of the Cambodian roadmap was broad and comprehensive, the Slovenian roadmap was narrowly focused (on pilot interventions) and detailed. With regards to the *format*, the Cambodian roadmap developed as a comprehensive set of policy recommendations towards a national strategic document (depicting a multi-faceted and multi-stakeholder process from evidence to policy); while in Slovenia, no comprehensive “roadmap” was developed, rather it took the shape of two study protocols for pilot-projects, offering an implementation plan (whilst the evaluation is ongoing), feeding into national developments (eyeing a more linear process from pilot to policy). In Belgium, the roadmap constitutes of a series of internal and external documents consisting out of an overview of evidence as rationale, descriptions of collaborative processes and targets, aligning with the adopted networking approach as well as the complex and fragmented health policy context. While there is no consensus-based definition of a roadmap within implementation science, all three “roadmaps” developed as part of SCUBY present evidence-driven, and stakeholder supported strategies and actions with relevant goals in relation to time. In terms of the *prioritized scale-up dimension*, the Cambodian roadmap has a strong focus on enabling and integrating essential services for T2D and HT (i.e., working on all scale-up dimensions but mostly focused on increasing *coverage and institutionalisation* of the WHO PEN program), while in Slovenia, where there are already high levels of integration/institutionalisation and coverage of integrated care, the roadmap took a strong focus on (technology-supported) task-shifting to improve inclusiveness of elderly and other vulnerable populations (i.e., *expanding the ICP* with aim to increase equitable coverage). The Belgium roadmap centres around a networking approach to facilitate dialogue and synergies between diverse stakeholders within a fragmented health care system (i.e., focused on producing change to support *integration or institutionalisation*).

Overall, as a learning, uni-dimensional scale-up roadmaps are not able to tackle complex realities, instead, while different scale-up dimensions were primarily focused on within the different countries, the roadmaps still captured other scale-up dimensions and acted to ‘fill the gap’, considering contextual needs. The final difference between roadmaps relates to the influence, *mandate, and power of the change team* in the case-study country. For example, in Cambodia, the National Institute of Public Health (NIPH) was a partner in the SCUBY consortium. In their advisory role to the Ministry of Health (MoH), the Cambodian roadmap contains a wide range of recommendations which supports national health system strengthening in line with their mandate. In Slovenia, the SCUBY consortium partner represents the largest Community Health Centre (ie, the Community Health Centre of Ljubljana) which also serves as a role-model, for scaling interventions that are deemed effective and feasible, for other health centres. Consequently, the roadmap developed by the Slovenian SCUBY team focussed on an operational approach (i.e., testing the feasibility of quality improvement interventions through pilot projects). Finally, in Belgium, the SCUBY partners are academic institutions (University of Antwerp and Institute of Tropical Medicine, Antwerp) with limited direct influence and mandate in the organisational or policy field. Hence, a networking approach emerged as an essential component of the roadmap highlighting the importance of science communication to various stakeholders working in the integrated care for chronic diseases field and subsequently creating synergetic partnerships. In summary, a final learning here is that the nature of the roadmap and its strategies can partly rely on the positionality and mandate of the partners involved in their development. Selecting partners carefully would be imperative for a roadmap to be adopted for impact.

## Discussion

In the SCUBY project, three evidence-based scale-up roadmaps for integrated care of T2D and HT across three distinct health systems were developed. The three roadmaps reflect differences in the historical context and current realities within the three distinct health systems, and partly, may be shaped by the expertise and proximity to power of the country-specific working group and extended network (combined, the change team) developing them [31]. Taking this into consideration, roadmaps can be considered complex interventions. In the SCUBY scale-up, multiple roadmap components and stakeholders interact, producing emergent effects which are different from the effects of the individual elements and actors within a socio-ecological system [32,33]. The roadmap interventions can change over time because of contextualisation and adaptation. Hence, these roadmaps should be considered a flexible and fluid set of country-specific strategies that evolve over time [34]. highlight the importance of renewing and regenerating complex interventions [14]. Recent implementation studies have therefore stressed the relevance of documenting modifications to evidence-based practices [35,36,37]. The scale-up roadmaps developed as part of SCUBY indeed emphasise and document the (continuous) adaptation as a result of a co-creative process. To our knowledge, evidence on the use of roadmaps – as a knowledge translation and mobilisation instrument – for scale up is limited [38,39,40,41,42,43]. Our thematic analyses on the roadmap content, identifying key elements, actions and strategies, as well as similarities and contextual differences can help inform other experiences on (the benefits of) roadmap usage. Hence, this paper offers a response to the need to better understand the various strategies on *how* to scale up [5,34]. Examining such strategies, policy plans, or ‘roadmaps’ is crucial to enhance scale-up efforts as well as chronic disease control and health system strengthening.

### Lessons learnt: proposing a conceptual spiral model for scale-up

**Figure 2:**
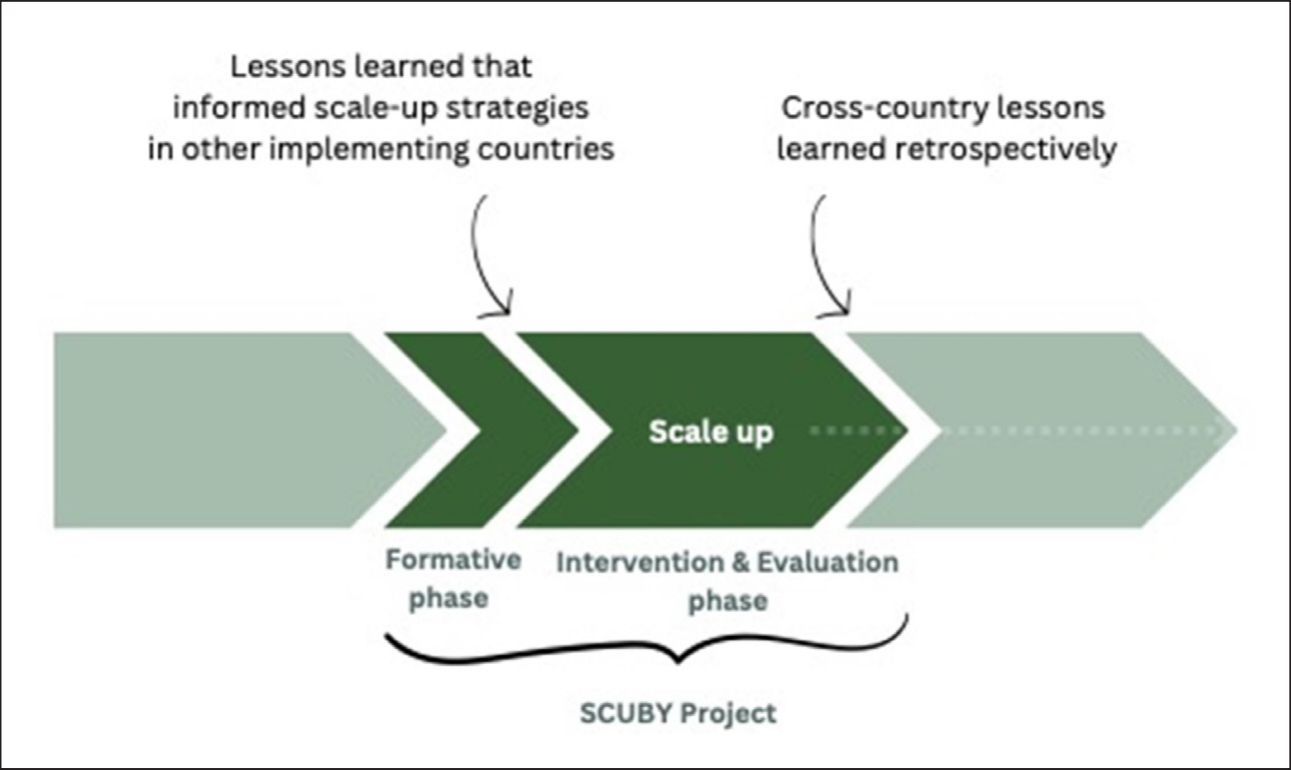
Cross-country lessons can be drawn prior to the roadmap development as well as retrospectively.

Reflections and lessons were shared at various exchange moments during the SCUBY project on what would constitute relevant and effective scale-up strategies or roadmaps. Overall, reciprocal learnings on the roadmap from the three country cases took place at two different stages, i.e., at the end of the formative phase and intervention phase, as illustrated by Figure 2.

In summary, the various reciprocal learnings on similarities and differences in scale-up roadmap’s content, team and format point to:

- Similar strategies with regards to task-shifting (to patients themselves, nurses and community health workers); strengthening monitoring and evaluation; and creating an enabling environment for ICP implementation.
- The importance of boundary spanning in the pursuit of ICP scale-up across all three contexts
- A variety in roadmap design: roadmaps can come in different formats, fitting with contextual needs and preferences of the change team
- Different scale-up priorities or dimensions (coverage, expansion, integration) which is given priority to
- A variety in mandates and hence the importance of carefully selecting the change team and scale-up partners given the influence of their mandate and positionality/ies
- With regards to implementation: developing a roadmap does not equal (partial) implementation or policy adoption. Within SCUBY, the scale-up roadmap is ever evolving and provides a complex perspective.

The reciprocal learnings *at the end of the formative phase* mostly involved the ones on dis/similar roadmap ‘content’, actions or strategies (a) and scale-up dimensions given priority to (d). *At the end of the project, retrospective learnings* made by the authors in concertation with the wider team included those on boundary spanning (b), and format or roadmap design (c) and mandates/positionality (e). The final lesson (f) relates to the extent of implementation and policy adoption; given that we use a complexity perspective in this project [10,44], the scale-up roadmap is constantly evolving, and hence, complex to report on outside of the scope of this study.

**Figure 3:**
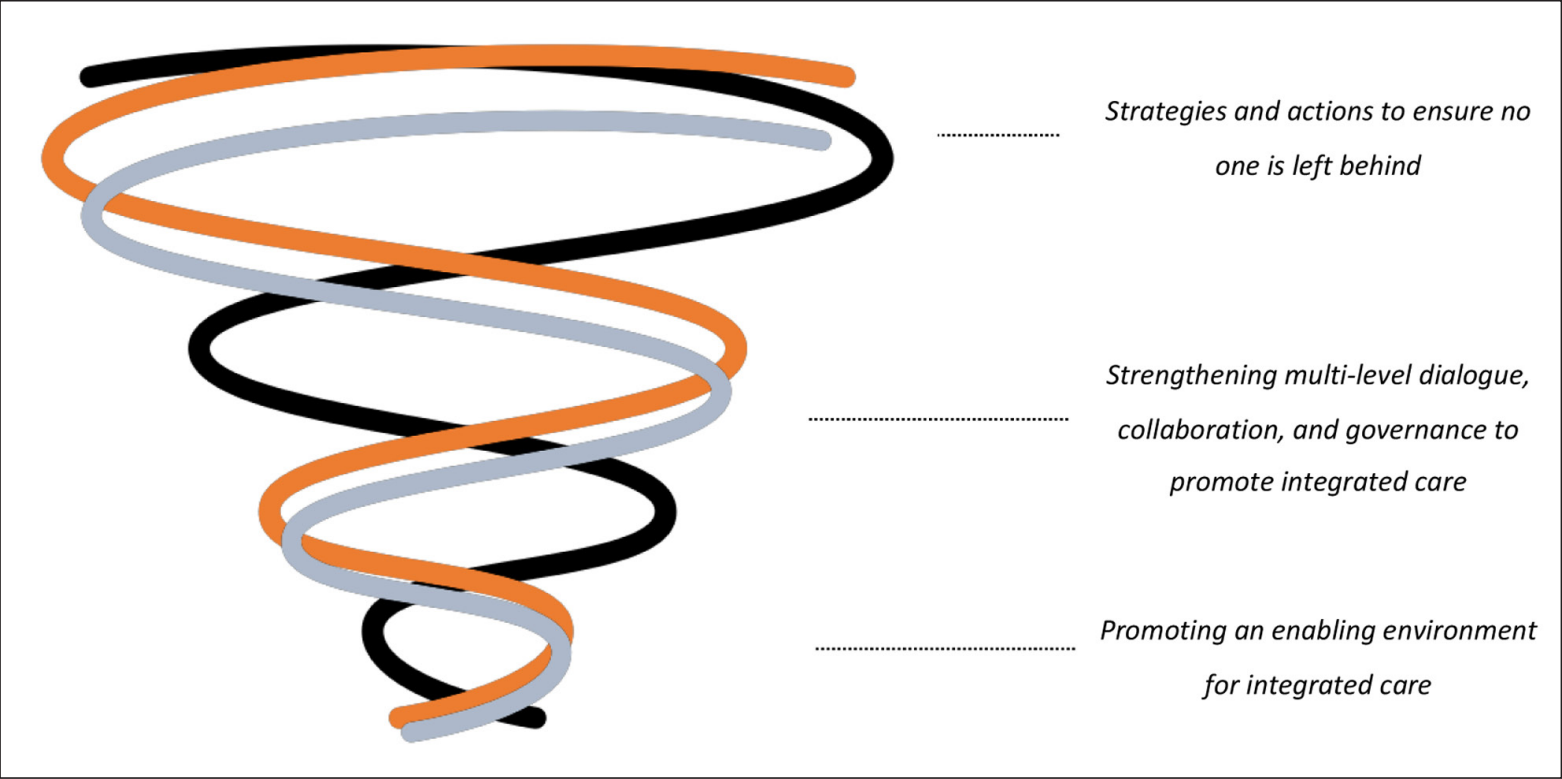
Conceptual model for scaling-up integrated care across health systems, aligned with the scale-up dimensions reported elsewhere [5], where (i) an enabling and elastic environment is a pre-requisite for sustainable care integration and expanding the package of care, (ii) subsequent dialogue is required to institutionalise integrated care within existing governance structures, to (iii) then adopt diversification strategies that focus on coverage of vulnerable populations to not be left behind.

In reflection of the reciprocal learning approach, we conceptualised the cross-country focal areas in scaling-up integrated care across these health systems as a spiral process from creating an enabling environment towards ensuring no one is left behind in Figure 3. We aligned this conceptual ‘spiral’ model with the scale-up dimensions [5], where (i) an enabling and elastic environment is a pre-requisite for sustainable care integration and *expanding* the package of care (key for health systems strengthening), (ii) subsequent dialogue is required to *institutionalise* integrated care within existing governance structures (through collaborative governance and policy-making), to (iii) then adopt diversification strategies that focus on *coverage* of vulnerable populations to not be left behind (i.e. strategies for health equity promotion). Relatedly, Cambodia’s roadmap is mostly focused on creating an enabling environment (i), Belgium’s roadmap adopted a network approach to enter dialogues with a broad variety of stakeholders (ii), while Slovenia’s pilot interventions focused on reaching vulnerable populations (iii). In that regard, the roadmap development and subsequent identified strategies need to be valued in the context of evolving practices, policies and their implementation (Figure 1). In Cambodia, in collaboration with NGOs and development partners, many initiatives are ongoing to *enable a supportive environment* by strengthening health care and broader economic development. Hence, amidst this, the Cambodian roadmap aimed to identify and synthesize critical yet synergistic strategies (e.g., human resources for health) and actions that can contribute to integrate PEN as a nationwide adopted strategy to establish standardized primary NCD prevention and care and further increasing its reach (coverage) through health system strengthening, including implementation of the revised (second version) PEN model in some health centres, a NGO-led diabetes peer support network (MoPoTsyo), NCD-training of public health care workers by the Ministry of Health, amongst others. Conversely, in Belgium, there is a wide array of disjointed strategies and policies that have been implemented at various levels, involving a broad range of stakeholders. Supporting mechanisms to span boundaries between these initiatives are often lacking. As a result, the Belgium roadmap focuses on addressing these structural gaps (integration) through *strengthening multi-level dialogues, collaboration, and governance*, specifically via three core roadmap actions, which included: supporting change management and task shifting; data harmonisation and monitoring; and health financing reform. Finally, in Slovenia, policies are well implemented and support integrated care organisation and multi-profile teams. Hence, the Slovenian roadmap subsequently focused on providing evidence to extend (expand) these services to vulnerable populations (i.e., rural, elderly) via patient empowerment and self-management, *ensuring no one is left behind*.

All three overarching scale-up strategies are relevant and ideally coinciding, but we found from our three study cases that countries are focused on different strategies (cf. scale-up dimensions given priority to), and therefore assume there are various levels of scale-up to achieve (starting from creating an enabling environment; e.g. local/partial health systems strengthening; to collaborative governance and health equity promotion). In reality an opted strategy may work or have effects across all these domains (or levels in the spiral model). Nevertheless, we found these useful as a general categorisation of overarching strategies to scale-up; in the way they align with what national governments are focusing most on in their scale-up efforts. Hence, also other countries with similar or dissimilar health system and policy contexts, may be benefitting from such conceptual scale-up model.

### Limitations

This study had some limitations. First, the COVID-19 pandemic halted much progress in developing and implementing the roadmaps for integrated care for chronic diseases due to other immediate priorities of care providers, decision makers and other stakeholders related to COVID-19 emergency responses. It affected the ‘policy window’ and critical juncture to move from development to implementation [45]. The implication thereof was that the roadmaps were developed in an extended period of time within a rapidly changing health landscape. However, irrespective of COVID-19, the SCUBY project window was relative short to develop, implement *and* evaluate the impact of the three roadmaps. A second limitation relates to the potential transferability of the roadmaps to other (similar) contexts. While we are confident that the roadmaps can be applied to scale-up integrated care package for HT and T2D in Cambodia, Slovenia, and Belgium, it is unclear to what extent these roadmaps can be generalised to other countries, and more specifically, to other types of health systems or health systems which have different pre-implementation characteristics. Nevertheless, we believe that the strengths of this research lie the reciprocal learning that was stimulated and the cross-country lessons that were drawn which are transferable. This warrant (self-)reflexivity on one’s power and role within scale-up efforts and on the implications of diverse and pre-existing cultures, practices, policies, histories, and political contexts. A final limitation relates to this lesson on self-reflexivity. We are aware that, to some extent, the roadmaps may reflect the SCUBY country change team’s positionality and power within their respective country to engage with, inform and influence essential stakeholders to shape the strategies and actions included in the roadmap. The latter reflects the realities in implementation science, which can both hinder and support progress towards scale-up. Irrespective, the three country cases present unique processes to roadmap development and implementation, from which one can learn about contextual differences and similarities to support scale-up through various strategies in these and other countries. As part of the roadmap development process, the respective country change teams have been able to gain boundary spanning skills and enter dialogues that can further assist the scale-up of integrated care. Future research and case studies can be conducted to further untangle the interactions between scale-up dimensions, and test and revise the conceptual spiral model for scale-up.

### Conclusion

As part of the SCUBY project, we were able to co-create, in close collaboration with stakeholders, three roadmaps for integrated T2D/HT care across three distinct health systems. The differences in strategies and actions in these roadmaps reflect differences in the historical context and current realities in each of the case study countries. Similar overarching strategies relate to creating an enabling environment for integrated care, facilitating dialogue to institutionalise integrated care in routine practice and optimizing resources through task-shifting to promote equitable access. There lies inherent value in exploring similarities and differences through a consortium approach while developing national or regional strategies to strengthen health systems.

## Additional Files

The additional files for this article can be found as follows:

10.5334/ijic.8618.s1 Supplementary file. Online supplement 1.

10.5334/ijic.8618.s2 Supplementary file. Online supplement 2.
