# Supplementary material for: Country-Specific Roadmaps for Scaling Up Integrated Care in Belgium, Slovenia, and Cambodia – Lessons Learned from the SCUBY Project
Source: Int J Integr Care. 2024 Dec 20;24(4):17. doi: 10.5334/ijic.8618 (PMC11661016; doi:10.5334/ijic.8618)
Supplement: Supplementary file. — Online supplement 1. [file ijic-24-4-8618-s1.pdf]

Online supplement 1

Table of contents

Country profiles **2**

References **4**

Roadmap elements according to ExpandNet Framework **6**

Country profiles

Overview of socio-economic situation, health system characteristics and NCD profile in the three study settings

	Cambodia	Slovenia	Belgium
**Governance**			
• Government type	Sovereign state, a unitary dominant-party parliamentary elective constitutional monarchy	Unitary parliamentary (post-socialist) democratic republic	Federal parliamentary democracy under a constitutional monarchy
• Country wealth	Lower-middle income	High income	High income
• Regional organisation	25 provinces (first-level administrative division), subdivided into 159 districts and 26 municipalities (second-level administrative divisions)	62 administrative districts or units as subdivisions of national government administration and 212 municipalities (including 12 urban municipalities) as sole bodies of local autonomy, with no intermediate level between municipalities and the Republic of Slovenia [1]	There are three levels of power in Belgium comprising the Federal authorities, Federated entities (three regions based on territory and three communities based on language – Dutch, French, and German) and local authorities (provinces and municipalities) [2]
**Socio-economic profile**			
• GDP/Capita in 2021 [3]	1,661.7 USD	29,200.8 USD	51,767.8 USD
• GDP growth rate in 2021 [4]	3.0%	8.1%	6.2%
• Income inequality (Gini index)	30.8 (2012) [5]	24.4 (2019) [6]	27.2 (2019) [6]
• Poverty rate [7]	17.7% (2012)	12.4% (2019)	14.1% (2019)
**Health system characteristics**			
• Health system organisation	Pluralistic–less regulated public health system and large yet little regulated private sector (important role of NGOs)	Centralised [1] public health system with growing regulated private sector	Federal system, with partially decentralised (fragmented) responsibilities, mainly private providers
• Organisation	102 ODs (each serving 100,000–200,000 people), 9 national hospitals, 25 provincial RHs, 89 district RHs (approx. one for each OD), and 1,205 health centres (covering 8,000–12,000 people) [8, 9]	Inpatient hospital care is provided by 30—mostly public—hospitals, while primary care is mostly provided by a network of 63 multidisciplinary community-based primary healthcare centres, owned and run by the municipalities [1, 10], in addition to private facilities contracted by the Health Insurance Institute	104 acute care hospitals, 60 psychiatric hospitals, 9 specialised or geriatric hospitals [2], 60 primary care zones in Brussels and Flanders for coordination, many solo GP practices (61% in 2018); 39% in group practices with other GPs; and 6% within capitation system [11]
• Health financing	State and large private	Societal (social	Societal (social

system	system	insurance)	insurance)
• Healthcare provision	Mixed provider system (large private system, with little regulation and enforcement especially at primary care level)	Mixed system of public-private providers, regulation of package and tariffs<br><br>Strong public primary care system	Mixed system of public and private providers (mainly private providers, regulation of package and tariffs with some degree of freedom)<br><br>Hospital-oriented<br><br>High supply & choice-oriented public system
• Primary care purchasing and payment	Public providers paid fixed salary, private providers FFS	Mixed payment of capitation/FFS for all	Providers mainly opt for FFS, but there is also a capitation-based remuneration system
• Embedding of primary care in community network	Variable: community-based workers throughout the country but links with primary care variable	Strong with a community-based nurse in primary care practice	Medium, recent reforms aim for population-based IC
• Health expenditure (USD/capita) in 2019 [12]	316.05 USD	3,629.01 USD	5,846.90 USD
• Health expenditure (% of GDP) in 2019 [13]	6.99%	8.52%	10.66%
• Number of physicians per 1,000 inhabitants [14]	0.2 (2014)	3.2 (2018)	6.0 (2019)
• Number of primary care physicians per 1,000 inhabitants [15]<br>•	n.d.	1.19 (2020)	0.62 (2020)
• Number of nurses per 1,000 inhabitants [16]	1.0 (2019)	10.2 (2018)	11.8 (2015)
**Demographics**			
• Total population [17]	16,487,000	2,079,000	11,539,000
• Population older than 65 (% of total population) [18]	5% (2021)	21% (2021)	20% (2021)
• Life expectancy at birth, total (years) [19]	70 (2020)	81 (2020)	81 (2020)
• Probability of premature mortality from NCDs	23%	11%	11%
• Percentage of deaths from NCDs [17]	68%	90%	86%

Note: The following abbreviations are used in the table: GDP = Gross Domestic Product, GP = General practitioner, IC = Integrated care, NCD = Non-communicable disease, n.d. = no data, NGO = Non-governmental organisation, OD = Operational District, RH = Referral hospital, USD = United States Dollar

References

1. European Observatory on Health Systems and Policies. Slovenia: health system review 2021. 2021 [1 April 2023]; Available from: https://eurohealthobservatory.who.int/publications/i/slovenia-health-system-review-2021.
2. European Observatory on Health Systems and Policies. Belgium: health system summary. 2021 [1 April 2023]; Available from: https://eurohealthobservatory.who.int/publications/i/belgium-health-system-summary.
3. World Bank. GDP per capita (current US$) | Data. World Bank Open Data; 2022 [1 April 2023]; Available from: https://data.worldbank.org/indicator/NY.GDP.PCAP.CD.
4. World Bank. GDP growth (annual %) | Data. World Bank Open Data; 2022 [1 April 2023]; Available from: https://data.worldbank.org/indicator/NY.GDP.MKTP.KD.ZG.
5. World Economics. Cambodia's Gini Year. 2021 [1 April 2023]; Available from: https://www.worldeconomics.com/Inequality/Gini-Year/Cambodia.aspx.
6. World Bank. Gini index | Data. World Bank Open Data; 2022 [1 April 2023]; Available from: https://data.worldbank.org/indicator/SI.POV.GINI?name_desc=false.
7. World Bank. Poverty headcount ratio at national poverty lines (% of population) | Data. World Bank Open Data; 2022 [1 April 2023]; Available from: https://data.worldbank.org/indicator/SI.POV.NAHC?name_desc=false.
8. Ministry of Health - Cambodia. National standard operating procedure for diabetes and hypertension management in primary care 2019. Phnom Penh: MoH; 2019 [1 April 2023]; Available from: https://niph.org.kh/niph/uploads/library/pdf/GL240_PEN-SOP_DM_and_HBP_1_APR_2019-EN.pdf.
9. Ministry of Health - Department of Planning and Health Information - Cambodia. Annual Health Sector Progress Report 2018 and Way Forwards for 2019. 2019 [25 April 2023]; Available from: http://moh.gov.kh/content/uploads/2017/05/2019_MoH-Final01-Low.pdf.
10. European Observatory on Health Systems and Policies. Slovenia: health system summary. 2021 [1 April 2023]; Available from: https://eurohealthobservatory.who.int/publications/i/slovenia-health-system-summary.
11. Detollenaere J, Christiaens W, Dossche D, Camberlin C, Lefèvre M, Devriese S. Barriers and facilitators for eHealth adoption by general practitioners in Belgium. Health Services Research (HSR) - KCE Reports 337. Brussels. : Belgian Health Care Knowledge Centre (KCE); 2020 [1 April 2023]; Available from: https://kce.fgov.be/en/publications/an-eq-5d-5l-value-set-for-belgium-how-to-value-health-related-quality-of-life/barriers-and-facilitators-for-ehealth-adoption-by-general-practitioners-in-belgium.
12. World Bank. Current health expenditure per capita, PPP (current international $) | Data. World Bank Open Data; 2022 [1 April 2023]; Available from: https://data.worldbank.org/indicator/SH.XPD.CHEX.PP.CD.
13. World Bank. Current health expenditure (% of GDP) | Data. World Bank Open Data; 2022 [cited 1 April 2023]; Available from: https://data.worldbank.org/indicator/SH.XPD.CHEX.GD.ZS.
14. World Bank. Physicians (per 1,000 people) | Data. World Bank Open Data; 2022 [cited 1 April 2023]; Available from: https://data.worldbank.org/indicator/SH.MED.PHYS.ZS.
15. OECD. Health Care Resources : Physicians by categories. OECD.Stat.; 2023 [1 April 2023]; Available from: https://stats.oecd.org/Index.aspx?QueryId=30173.

16. World Bank. Nurses and midwives (per 1,000 people) | Data. World Bank Open Data; 2022 [cited 1 April 2023]; Available from: https://data.worldbank.org/indicator/SH.MED.NUMW.P3.
17. World Health Organization. Noncommunicable Diseases Progress Monitor 2022. Geneva: World Health Organization; 2022 [1 April 2023]; Available from: https://www.who.int/publications/i/item/9789240047761.
18. World Bank. Population ages 65 and above (% of total population) | Data. World Bank Open Data; 2022 [cited 1 April 2023]; Available from: https://data.worldbank.org/indicator/SP.POP.65UP.TO.ZS.
19. World Bank. Life expectancy at birth, total (years) | Data. World Bank Open Data; 2022 [cited 1 April 2023]; Available from: https://data.worldbank.org/indicator/SP.DYN.LE00.IN?name_desc=false.

Roadmap elements according to ExpandNet Framework

Overview of different key roadmap elements for each country, stratified by the ExpandNET framework' strategies for scale-up (World Health Organization), and dimensions of scale-up.(van Olmen et al, Glob Health Action. 2020)

	**Cambodia**			**Slovenia**			**Belgium**		
	Horizontal (coverage)	Vertical (integration)	Diversification (expanding)	Horizontal (coverage)	Vertical (integration)	Diversification (expanding)	Horizontal (coverage)	Vertical (integration)	Diversification (expanding)
Organisational processes	- Strengthening the workflow of second-version PEN at the operational district level	- Revising / updating the components of ICP<br>- Integrating the second-version PEN with other vertical programs<br>- Ensuring appropriate staff/staff capacity / skills-mix through practical training on T2D & HT care (on-site training), including nurses and midwives.	- Adding community-based intervention to ICP		- An intra-team collaboration project: developing clinical pathways of patients for better team management	- Community-based education programme	- Expansion of care package: Scaling care pathway on heart failure.	- Study alternative financing models in primary care<br>- Stronger position of nurse practitioners within primary care to facilitate integration of care pathways.	
Stakeholder engagement, dissemination, and advocacy		- Strengthening leadership and management of human resources for health at the operational district and health centre level		- A group education programme by patients (patients as educators)				- Better chronic care by general practitioners through training	
Cost/resource	- Increasing	- Reinforcing the capacity of staff		- An m-health intervention		- An m-health intervention		- Human	

mobilisation	coverage of second-version PEN in primary healthcare<br>- Strengthening the essential medicine supply system<br>- Increasing the investment in T2D and HT<br>- Reducing financial burden to T2D and HT patients	in managing medicine inventories		to support and empower patients (telemedicine)		to support and empower patients (telemedicine)		resource management: Budget for nurse in primary care team<br>- Budget for chronic care that stimulates quality	
Monitoring and evaluation		- Strengthening Monitoring and evaluation						- Monitoring of chronic care indicators in Primary Care Zones<br>- Monitoring care organisation at practice level	

HT, Hypertension; ICP, Integrated Care Package; PEN, Package of Essential Interventions; T2D, Type 2 Diabetes
